# Study on the impact of learning engagement on the subjective wellbeing of empty nesters in rural China

**DOI:** 10.3389/fpsyg.2024.1497528

**Published:** 2024-12-11

**Authors:** Ruirang Zeng, Huiqin Shi, Huiqi Wu, Lixin Sun

**Affiliations:** ^1^Faculty of Maritime and Transportation, Ningbo University, Ningbo, China; ^2^College of Teacher Education, Ningbo University, Ningbo, China

**Keywords:** learning engagement, rural empty nesters in China, subjective wellbeing, elderly education, social support

## Abstract

With the rapid development of China's socio-economic landscape and shifts in population structure, rural empty nesters have increasingly become a focal point of social concern. Compared to their urban counterparts, rural empty nesters face more life challenges and psychological stress, making their subjective wellbeing a significant issue. This study explores the impact of learning engagement on the subjective wellbeing of rural empty nesters in China, aiming to provide theoretical support and policy recommendations to enhance their wellbeing. Using a random sampling method, the study focused on elderly learners aged 50 and above from counties within Z Province, N City, where elderly learning is relatively concentrated. Data were processed using SPSS 27.0 software. The findings indicate that there is a significant difference in subjective wellbeing between rural empty nesters and rural non-empty nesters, with rural empty nesters exhibiting lower subjective wellbeing and more passive involvement in learning activities. Learning engagement has a significant effect on improving the subjective wellbeing of rural empty nesters, with a notable positive correlation between learning engagement dimensions and subjective wellbeing. Compared to rural non-empty nesters, learning engagement has a more pronounced positive effect on the subjective wellbeing of rural empty nesters.

## 1 Introduction

The proportion of empty nesters among China's elderly population has now exceeded half, and in most urban and rural areas, this ratio surpasses 70%. A significant number of elderly individuals do not live with their children or other family members, facing numerous challenges related to aging at home (Interface News, 2022). The living conditions of rural empty nesters impact social harmony and stability; thus, helping them spend their later years peacefully and enhancing their wellbeing is essential for building a harmonious society. Subjective wellbeing refers to an individual's overall assessment of their quality of life based on internal standards, encompassing satisfaction with life and its various aspects, leading to a psychological state characterized by predominant positive emotions (Diener et al., 1999). It is also a key indicator of whether a country or region is experiencing positive aging (Tovel and Carmel, 2014). With the acceleration of urbanization and the migration of rural labor to cities, the proportion of left-behind elderly is gradually increasing (Wei, 2024). Rural empty nesters often face issues such as inadequate social support systems and need to improve their quality of life and wellbeing through alternative means. Learning participation, as an active form of elderly engagement, can offer rural empty nesters new lifestyles and opportunities for social interaction. This study investigates the relationship between learning participation and the subjective wellbeing of rural empty nesters in China, as well as the differences in the impact of various types of learning participation on their wellbeing, aiming to provide theoretical support and policy recommendations for enhancing the subjective wellbeing of rural empty nesters.

## 2 Literature review

### 2.1 Research on rural empty nesters

The concept of “empty nest” was first introduced by Sorokin, Zimmerman, and Galipin in 1931, defining “empty nesters” as elderly individuals living either with their spouse or alone. In 1946, Duvall expanded this concept within the context of his well-known family life cycle model, which includes eight stages: the childless couple stage, the expanded family stage, the preschool family stage, the school-age family stage, the family with adolescent children stage, the middle-aged parents stage, and the elderly family stage (Duvall, 1988). Relevant scholars have defined the concept of empty nesters from various perspectives, such as the duration of separation from children (Lv and Feng, 2018) and whether children live with the elderly (Zong et al., 2018). Logan et al. (1998), from the perspective of living arrangements, defined “empty nesters” as elderly individuals living alone or with their spouse. In 1986, Reuben Hill further elaborated on the concept of “empty nesters” from the perspective of “empty nest families,” suggesting that “empty nest families” refer to the post-parental empty nest stage (Abdullah and Wolbring, 2013). Although scholars both domestically and internationally have different views on the definition of “empty nesters,” they share a common understanding: regardless of the stage, “empty nesters” refer to a state where the children are no longer living with the elderly, and the household consists only of the couple. In this study, “empty nesters” refers to elderly individuals aged 50 and above who have no children or whose children are not living with them, residing alone or with a spouse in rural areas. This specifically includes three types: living alone, living with a partner, and residing in a nursing home.

Rural empty nesters have attracted increasing research attention due to their unique social structure and living conditions, with studies focusing on their physical and mental health, quality of life, social support, and retirement models (Gao et al., 2022). Regarding the causes of empty nesting among the elderly, with economic development and frequent rural-to-urban migration, young family members leaving for work has led to changes in household decision-making power. This shift has gradually reduced the status and support of the elderly within the family, thereby contributing to the formation of empty nest elderly in rural areas (Giles and Mu, 2007). Survey results from van de Walle (2019) show that the quality of life of the elderly is significantly correlated with the financial support provided by their children. In other words, when children provide more financial assistance, the quality of life of the elderly improves. However, a consequence of becoming an empty nester is the emergence of psychological health issues (Tomaka et al., 2006). Research has found that these empty nesters often face challenges such as health issues, psychological stress, and financial difficulties (Chen and Shen, 2020). In terms of physical health, the migration of rural children to cities significantly deteriorates the self-rated health of empty nesters and increases the likelihood of chronic diseases (Liu, 2022). Regarding mental health, a considerable number of rural elderly, especially empty nesters, are in a state of psychological deprivation, lacking companionship, and experiencing low self-efficacy, life satisfaction, and happiness (Wang and Wang, 2021). Sun et al. (2011) suggest that the long-term absence of children deepens empty nesters' attachment to their children, which can adversely affect their mental state. Furthermore, the primary reason for the loneliness experienced by rural empty nesters is the lack of social support (Zhang, 2023; Dykstra, 2009). Kang and Luo (2022) found through visits to a typical “empty nest” rural area in central Sichuan that these elderly individuals are often in a disadvantaged position regarding social support, facing issues such as lack of care, emotional loneliness, and spiritual desolation. Consequently, some rural empty nesters are compelled to shift their retirement model from the traditional family care model to a primarily self-care model with supplementary family care (Guo and Liu, 2023), opting for community mutual aid retirement models. In summary, under the new demographic structure, with shrinking family sizes and social class differentiation, the disadvantaged position of rural empty nesters continues to strengthen.

Based on this, this study proposes Hypothesis 1: Compared to rural non-empty nesters, rural empty nesters have lower physical and mental health and wellbeing.

### 2.2 Research on the factors affecting the subjective wellbeing of rural empty nesters

The factors influencing the subjective wellbeing of rural empty nesters can be categorized into personal factors, family factors, and social factors. Firstly, personal factors include physical health, age, gender, economic income, and education level. Tobiasz-Adamczyk and Zawisza (2017) used a simplified version of the national happiness index (DRM) to study subjective wellbeing among rural empty nesters in Poland, finding that an increase in chronic diseases was associated with a decrease in subjective wellbeing. Wang et al. (2017) found that elderly individuals with good physical health scored significantly higher in positive emotions, overall happiness, and life satisfaction compared to those with chronic diseases, who scored higher in negative experiences. Additionally, rural empty nesters with religious beliefs reported lower levels of subjective wellbeing (Feng and Luo, 2015).

Secondly, from a family perspective, factors such as having a spouse and intergenerational support are crucial for subjective wellbeing. Family plays a vital role in providing emotional comfort to empty nesters (Stillwell and Warnes, 1995; Cloutier-Fisher et al., 2011). Barron et al. (1994) found that elderly individuals living alone generally feel more lonely due to the lack of warm family relationships. A complete family plays a significant role in maintaining elderly individuals' mental health, while widowed elderly often experience severe negative emotions. Okun et al. (1990) noted that widowhood results in the loss of primary family care sources, exacerbating their economic, mental, and psychological conditions. Guo (2023), using the Logit model to analyze the factors affecting subjective wellbeing among 1,991 rural empty nesters, found that intergenerational support significantly affects the subjective wellbeing of those living alone. Moreover, excessive distance between children and parents negatively impacts the elderly's mental health (Benjamin et al., 2000).

Finally, social factors include living environment, interpersonal relationships, and social support. Long et al. (2022) found a positive correlation between subjective wellbeing and social support based on a survey of 494 rural empty nesters in Jiangxi Province, China. Xing et al. (2010) found a correlation between interpersonal relationships and elderly subjective wellbeing, noting that friendlier neighborhood relations are associated with higher life satisfaction (Li and Zhang, 2012). Research indicates that increasing peer interactions among empty nesters and enhancing social support from peers can alleviate feelings of loneliness (Andrews, 2020) and may even benefit disease recovery (Powell, 2012).

### 2.3 Study on the relationship between subjective wellbeing and learning participation among rural empty nesters

Research on the impact of learning participation on the wellbeing of rural empty nesters is limited both domestically and internationally, primarily consisting of descriptive studies showing its effects and a few empirical studies on specific activities (such as reading and sports). Firstly, Tong (2017) noted that attending elderly universities and taking courses based on actual needs can not only address the low cultural level of rural empty nesters but also fulfill many elderly individuals' “dreams of schooling.” Research shows that guiding public platforms, people's groups, and social institutions to help rural empty nesters with learning plans and providing platforms for social activities can help them break out of isolation and alleviate feelings of loneliness, making their later years more meaningful (Zhang, 2023). Liu et al. (2024) studied the role of elderly community groups in enhancing the wellbeing of rural empty-nest elderly individuals. They pointed out that elderly education classes not only assist older adults in applying knowledge to improve their lives but also effectively increase their sense of happiness. In the study of the correlation between learning participation and subjective wellbeing, Fan (2009) further pointed out that the six internal elements of participating in learning activities—namely, the enjoyment of learning, achievement motivation, challenge and difficulty, level of engagement, self-actualization, and learning atmosphere—are significantly positively correlated with the overall score of subjective wellbeing. From an empirical research perspective, Bai (2017) explored the mediating role of aging attitudes. The study found that there are significant correlations among the three variables of participation in learning activities, aging attitudes, and subjective wellbeing. Additionally, social loss and psychological gains have a significant mediating effect between participation in learning activities and subjective wellbeing. Dench and Regan (2000) showed that among 336 surveyed elderly learners, 80% experienced significant improvements in self-perception and life enjoyment and satisfaction. Du et al. (2016) investigated the impact of reading therapy on the subjective wellbeing of elderly individuals aged 75–80 residing in nursing homes in Tianjin. They conducted observations and recorded the scales used to assess the psychological and cognitive abilities of the elderly before and after the intervention. The results of the experiment indicated that reading therapy can enhance the subjective wellbeing of the elderly. Additionally, Yang and Xu (2024) found that participation in learning and other recreational activities can enhance elderly individuals' subjective wellbeing. However, excessively frequent participation in a single type of recreational activity may not improve subjective wellbeing as effectively as less frequent participation. For instance, rural elderly who engage in learning activities occasionally or regularly tend to have higher wellbeing compared to those who participate in such activities very frequently. Regarding specific learning activities, Kawakami et al. (2017) discovered that Japanese empty nesters experience improved positive emotions and subjective wellbeing when watching live sports events. Pan et al. (2018) suggested that indirect sports participation, such as watching matches, positively affects subjective wellbeing and is directly proportional. Escuder-Mollón et al. (2014) argued that engaging in learning activities can enhance the quality of life and increase happiness among the elderly. Su et al. (2022) found that the depression levels of empty-nest elderly individuals are higher than those of non-empty-nest elderly individuals. However, social participation can significantly reduce the depression of empty-nest elderly individuals, thereby enhancing their sense of wellbeing. For rural empty nesters, participating in learning activities helps reduce loneliness through social interaction, promotes physical and mental health, delays cognitive decline, boosts confidence and self-esteem through continuous learning opportunities, and ultimately enhances subjective wellbeing.

Boshier and Collins (1985) developed the Educational Participation Scale (EPS), which is widely used in adult learning surveys globally. The scale aims to systematically assess adult learners' motivation to learn, specifically including multiple aspects such as goal orientation, learning orientation, and activity orientation. It provides an important tool for researching adult learning, especially in evaluating the multidimensional characteristics of learning motivation, which has high scientific value. In addition, Fredricks et al. (2004) proposed a learning participation model based on three dimensions: behavior, cognition, and emotion. They argue that learning participation is not only reflected in the behavioral dimension, but also involves deep cognitive and emotional engagement, emphasizing the multidimensional interaction during the learning process. This model has provided a theoretical framework for subsequent research on learning participation and has influenced many scholars' research directions. Based on this theory, many researchers have designed self-developed questionnaires to measure different dimensions of learning participation. For example, Liu and Guo (2008), in her study, referred to Fredricks' model of learning participation and, combined with the actual situation of Chinese middle school students, created a questionnaire to assess students' learning behaviors, cognitive engagement, and emotional participation. Her study provided a new perspective on measuring student learning participation, especially through an in-depth exploration of the mediating role of school climate and wellbeing. In empirical research, Fan (2009) explored the relationship between internal factors of learning participation and the subjective wellbeing of older adults, finding significant positive correlations between factors such as learning enjoyment, learning environment, learning investment, the level of challenge, achievement motivation experiences, and self-actualization. These findings further reveal the positive impact of learning participation on the wellbeing of adults, especially older individuals. Furthermore, Bru et al. (2019) developed the EVS D scale from the perspective of learning motivation. The scale includes four subscales: behavioral participation, behavioral dissatisfaction, emotional participation, and emotional dissatisfaction, aimed at measuring both behavioral and emotional participation in learning. This scale extends the measurement framework of learning participation, particularly in its dual dimensions of emotion and behavior, providing new methods for studying learners' emotional engagement. Based on the research findings of these previous studies, Li (2020) and Zhang (2022) further explored learning participation in older adults, proposing that learning participation can be categorized into three core dimensions: learning environment, learning experience, and learning investment. Their research, which integrates the multidimensional measurement framework of learning participation, expands the understanding of learning participation in different groups. This study will continue to use the classification proposed by Li and Zhang, taking learning environment, learning experience, and learning investment as the core dimensions of learning participation, in order to provide theoretical and empirical support for further exploring the relationship between learning participation and older adults' subjective wellbeing.

Based on this, the paper proposes Research Hypotheses 2 and 3:

Hypothesis 2: Learning participation can enhance the subjective wellbeing of rural empty nesters in China.

Hypothesis 3: The positive impact of learning participation on the subjective wellbeing of rural empty nesters in China is greater than that on non-empty nesters in rural areas.

## 3 Research design

### 3.1 Sample selection

To further reveal the mechanism through which learning participation affects the subjective wellbeing of rural empty-nester elderly, this study employed a random sampling method. The research focused on elderly individuals aged 50 and above who participate in senior learning programs in the districts and counties under Z Province, N City, where elderly learners are relatively concentrated. An electronic questionnaire was prepared, and prior to the survey, participants were informed about the purpose of the study and their consent was obtained before distribution. The electronic questionnaires were randomly distributed through educational institutions such as senior universities, community colleges, and adult schools. A total of 1,350 questionnaires were collected, of which 1,234 were valid, resulting in a high valid response rate of 94.9%. The final sample composition was as follows: 792 responses from rural empty-nester elderly (64.20%) and 442 responses from rural non-empty-nester elderly (35.80%) (see Table 1).

**Table 1:** Demographic variables statistics.

**Variable**	**Empty-nester (*n* = 792)**	**Non-empty-nester (*n* = 442)**	**Total (*n* = 1,234)**
Total individuals	792 (64.2%)	442 (35.8%)	1,234
**Age**			
50–59	259 (21.0%)	225 (18.2%)	484 (39.2%)
60–69	378 (30.6%)	178 (30.6%)	556 (45.1%)
70–79	146 (11.8%)	39 (3.2%)	185 (15.0%)
Over 80	9 (0.7%)	0 (0.0%)	9 (0.7%)
**Gender**			
Male	117 (9.5%)	52 (4.2%)	169 (13.7%)
Female	675 (54.7%)	390 (31.6%)	1,065 (86.3%)
**Marital status**			
With spouse	718 (58.2%)	395 (32.0%)	1,113 (90.2%)
Without spouse	74 (6.0%)	47 (3.8%)	121 (9.8%)
**Monthly income**			
Below 1,000	27 (2.2%)	13 (1.1%)	40 (3.2%)
1,000–2,000	100 (8.1%)	65 (5.3%)	165 (13.4%)
2,000–4,000	336 (27.2%)	199 (16.1%)	535 (43.4%)
Above 4,000	329 (26.7%)	165 (13.4%)	494 (40.0%)
**Education level**			
Elementary or below	33 (2.7%)	19 (2.7%)	52 (4.2%)
Junior high	284 (23.0%)	131 (10.6%)	415 (33.6%)
Senior high (including vocational)	310 (25.1%)	194 (15.7%)	504 (40.8%)
Associate degree	111 (9.0%)	69 (5.6%)	180 (14.6%)
Bachelor's or higher	54 (4.4%)	29 (2.4%)	83 (6.7%)
**Living situation**			
Living alone	90 (7.3%)	0 (0.0%)	90 (7.3%)
Living with spouse only	702 (52.6%)	0 (0.0%)	702 (52.6%)
Living with children	0 (0.0%)	110 (8.9%)	110 (8.9%)
Living with spouse and children	0 (0.0%)	331 (26.8%)	331 (26.8%)
Living in care facility	0 (0.0%)	1 (0.1%)	1 (0.1%)

### 3.2 Variable definitions and coding

Based on a comprehensive consideration of elderly learners' learning behaviors and engagement, the independent variable in this study is learning participation, which includes three dimensions: learning atmosphere, and learning experience, learning investment.

Learning Atmosphere includes elderly learners' perceptions and experiences regarding the teaching ability of senior university instructors, the teacher-student relationship, and the relationships among learners.

Learning Experience includes learning interest, learning initiative, learning concentration, and the importance of learning. These aspects are coded from “1—Strongly Disagree” to “6—Strongly Agree.”

Learning Investment consists of time investment, number of courses, and monetary investment. Time investment is coded as 1–4, representing >1, 1–5, 6–10, and over 10 years, respectively. Monetary investment is coded as 1–5, representing 0, 1–50, 50–100, 100–200, and over 200 yuan, respectively. Number of courses is coded as 1–4, representing 1 course, 2 courses, 3 courses, and 4 or more courses, respectively.

After standardizing the above data, the reliability of the independent variable questionnaire was tested, with a Cronbach's α of 0.854, indicating good overall reliability with statistical significance. The results of the confirmatory factor analysis show a good model fit: χ^2^/df = 1.762, CFI = 0.99, TLI = 0.986, GFI = 0.972, and RMSEA = 0.041, indicating that the structural validity of the scale is satisfactory.

The dependent variable in this study is the subjective wellbeing of the elderly. The scale design was based on the “Subjective Wellbeing Scale for Urban Residents (Brief Version)” from China. While maintaining the original structure, dimensions, and scoring methods of the questionnaire, adjustments were made to some items based on field surveys. The elderly's subjective wellbeing was categorized into three dimensions: physical and mental health experience, adaptation and satisfaction experience, and self-development experience.

Physical and Mental Health Experience includes indicators such as psychological health experience, physical health experience, and emotional balance experience.

Self-Development Experience includes indicators such as goal value experience, growth and progress experience, and self-acceptance experience.

Adaptation and Satisfaction Experience primarily includes indicators such as interpersonal adaptation experience, family atmosphere experience, contentment and abundance experience, and social confidence experience.

The scale consists of 10 measurement items, scored on a 6-point scale from “1—Strongly Disagree” to “6—Strongly Agree.” Higher scores indicate greater subjective wellbeing of the respondents.

After standardization, the reliability and validity testing results of the dependent variable questionnaire showed a Cronbach's α of 0.882, indicating good overall reliability. The confirmatory factor analysis results demonstrated a good model fit: χ^2^/df = 1.157, CFI = 0.984, TLI = 0.997, GFI = 0.997, and RMSEA = 0.019, indicating that the structural validity of the scale is satisfactory.

The demographic variables in this study are divided into two parts: individual characteristics and family characteristics. Individual characteristics include variables such as age, gender, education level, and monthly income. Family characteristics include variables such as marital status and living conditions (specific values and coding details are provided in Table 2).

**Table 2:** Variable coding and explanation.

**Variable**		**Value**	**Explanation**
Age		1–4	1: 50–59 years old; 2: 60–69 years old; 3: 70–79 years old; 4: 80 years and above
Gender		1–2	1: male; 2: female
Marital status		1–2	1: with spouse; 2: without spouse
Living situation		1–5	1: living alone; 2: living only with spouse; 3: living with children; 4: living with spouse and children; 5: living in a care facility
Monthly income		1–4	1: below 1,000 RMB; 2: 1,000–2,000 RMB; 3: 2,000–4,000 RMB; 4: Above 4,000 yuan RMB
Education level		1–5	1: primary school and below; 2: junior high school; 3: high school (including technical secondary school, vocational high school); 4: associate degree; 5: bachelor's degree and above
Subjective wellbeing	Physical and mental health experience	1–6	From 1 “strongly disagree” to 6 “strongly agree”, with higher scores indicating greater subjective wellbeing.
	Adaptation and satisfaction experience		
	Self-development experience		
Learning engagement	Learning atmosphere	1–6	From 1 “strongly disagree” to 6 “strongly agree”, with higher scores indicating higher levels of learning Engagement among the elderly.
	Learning experience		
	Learning investment		

Finally, the overall homogeneity reliability of the questionnaire is α = 0.91, with coefficients for each dimension and the total scale ranging from 0.80 to 0.93. The composite reliability for all variables is >0.7, the Average Variance Extracted (AVE) is >0.5, and the square root of the AVE is greater than the correlation coefficients between dimensions. This indicates that the reliability and validity of the overall scale meet the research requirements.

### 3.3 Research approach

This study uses SPSS 27.0 as the research tool. First, descriptive statistical analysis and difference tests are conducted to analyze the differences in subjective wellbeing and dimensions of learning participation among rural empty-nest elderly based on demographic variables. Next, an independent samples *T*-test is used to determine if there are significant differences in subjective wellbeing and learning participation dimensions between rural empty-nest elderly and rural non-empty-nest elderly. Subsequently, correlation analysis is employed to identify the relationship between learning participation and subjective wellbeing among rural empty-nest elderly. Finally, based on the correlation analysis, hierarchical regression is used to examine the mechanisms through which learning participation impacts the subjective wellbeing of rural empty-nest elderly and to identify specific differences in the impact of learning participation on the subjective wellbeing of both rural empty-nest and non-empty-nest elderly groups.

### 3.4 Analysis results

#### 3.4.1 Descriptive statistical analysis and difference testing

Research data indicate (see Table 3) that there are significant differences in subjective wellbeing across different genders, age groups, and education levels. Specifically, female elderly individuals have higher levels of happiness than males. Among age groups, those aged 80 and above report the lowest subjective wellbeing. No significant differences in subjective wellbeing are found across different monthly income levels, suggesting that income does not affect elderly individuals' subjective wellbeing. Additionally, subjective wellbeing does not differ significantly among elderly individuals with different marital statuses.

**Table 3:** Descriptive statistics and difference testing results for variables.

	**Category**	**Subjective wellbeing**	**Learning atmosphere**	**Learning experience**	**Learning engagement**
Marital status	With spouse	5.44 ± 0.83	5.45 ± 0.79	5.43 ± 0.82	3.89 ± 0.64
	Without spouse	5.46 ± 0.86	5.50 ± 0.83	5.50 ± 0.87	3.95 ± 0.76
	*t*	−0.24	−0.65	−0.89	−0.79
Gender	Male	5.16 ± 0.98	5.23 ± 0.87	5.23 ± 0.97	3.89 ± 0.68
	Female	5.48 ± 0.80	5.49 ± 0.77	5.47 ± 0.79	3.90 ± 0.64
	*F*	22.08^***^	15.13^***^	13.12^***^	0.01
Age	50–59 years	5.45 ± 0.79	5.48 ± 0.72	5.44 ± 0.78	5.48 ± 0.72
	60–69 years	5.44 ± 0.86	5.45 ± 0.81	5.46 ± 0.83	5.45 ± 0.81
	70–79 years	5.45 ± 0.85	5.41 ± 0.84	5.44 ± 0.86	5.41 ± 0.84
	80 years and above	4.46 ± 1.19	4.33 ± 1.55	4.27 ± 1.36	4.33 ± 1.55
	*F*	4.19^**^	6.50^***^	6.28^***^	11.97^***^
Monthly income	Below 1,000 RMB	5.31 ± 0.95	5.30 ± 1.14	5.22 ± 1.09	3.50 ± 0.73
	1,000–2,000 RMB	5.52 ± 0.82	5.48 ± 0.86	5.51 ± 0.84	3.67 ± 0.70
	2,000–4,000 RMB	5.45 ± 0.83	5.48 ± 0.75	5.48 ± 0.80	3.85 ± 0.62
	Above 4,000 RMB	5.41 ± 0.83	5.42 ± 0.77	5.40 ± 0.82	4.05 ± 0.61
	*F*	1.14	1.02	2.13^*^	23.66^***^
Education level	Elementary school or below	5.39 ± 1.05	5.33 ± 1.07	5.25 ± 1.18	3.51 ± 0.78
	Junior high school or below	5.53 ± 0.79	5.51 ± 0.79	5.52 ± 0.78	3.85 ± 0.68
	High school (including secondary technical, vocational)	5.50 ± 0.73	5.52 ± 0.70	5.51 ± 0.71	3.94 ± 0.62
	Associate degree	5.22 ± 0.96	5.25 ± 0.89	5.20 ± 0.93	3.98 ± 0.61
	Bachelor's or higher	5.12 ± 1.08	5.29 ± 0.89	5.22 ± 1.01	3.92 ± 0.53
	*F*	8.10^***^	5.59^***^	7.95^***^	6.46^***^

^*^p < 0.05, ^**^p < 0.01, ^***^p < 0.001.

In terms of elderly learning participation, there are significant differences in learning atmosphere, learning experience, and learning engagement across different education levels and age groups. However, no significant differences are found in learning engagement between different genders. Elderly individuals with varying monthly incomes show significant differences only in learning engagement, while no significant differences are observed in learning atmosphere, learning experience, or learning engagement among those with different marital statuses.

#### 3.4.2 Independent sample t-test analysis of learning participation and subjective wellbeing in rural empty-nest and non-empty-nest elderly groups

To further clarify the differences between rural empty-nest and non-empty-nest elderly groups in terms of learning participation and subjective wellbeing, an independent samples *t*-test was conducted on the sample data using SPSS (as shown in Table 4). The study data indicate that, on average, the rural empty-nest elderly group scores lower than the rural non-empty-nest elderly group in the dimensions of learning atmosphere, learning experience, and learning investment. Additionally, there are significant differences between the two groups in all three dimensions of subjective wellbeing, with the rural empty-nest elderly group showing lower levels in each aspect compared to the rural non-empty-nest elderly group. Overall, there is a significant difference in subjective wellbeing between the rural empty-nest and non-empty-nest elderly groups, with the former exhibiting lower wellbeing and more passive engagement in learning activities. Hypothesis 1 is thus validated.

**Table 4:** Independent sample *T*-test.

**Variable**		**Empty-nest**		**Non-empty-nest**		***T*-value**	**Sig**.
		**Mean**	**Standard deviation**	**Mean**	**Standard deviation**		
Learning engagement	Learning atmosphere	5.44	0.84	5.48	0.70	0.964	0.34
	Learning experience	5.42	0.86	5.45	0.76	1.198	0.23
	Learning investment	3.90	0.66	3.90	0.63	−0.036	0.97
Subjective wellbeing	Physical and mental health experience	5.34	0.96	5.47	0.80	2.601	0.01
	Self-development experience	5.38	0.93	5.50	0.78	2.493	0.01
	Adaptation and satisfaction experience	5.47	0.85	5.57	0.70	2.200	0.03

### 3.5 Correlation analysis of learning participation and subjective wellbeing among rural empty-nest elderly

To investigate the correlation between learning participation and subjective wellbeing among rural empty-nest elderly, Pearson correlation analysis was conducted using SPSS (see Table 5). The results indicate that all dimensions of learning participation among the rural empty-nest elderly are positively correlated with physical and mental health experience, self-development experience, adaptation and satisfaction experience, and overall subjective wellbeing, with the correlations being significant (*p* < 0.01). Specifically, learning engagement shows a low correlation with the subjective wellbeing of the rural empty-nest elderly, learning atmosphere shows a moderate correlation, and learning experience shows a high correlation. This suggests that participating in learning activities can significantly enhance the subjective wellbeing of rural empty-nest elderly.

**Table 5:** Correlation analysis of learning participation and dimensions of subjective wellbeing among rural empty-nest elderly (*n* = 742).

**Variable**	**1**	**2**	**3**	**4**	**5**	**6**	**7**
1. Learning experience	1						
2. Learning atmosphere	0.867^***^	1					
3. Learning investment	0.126^**^	0.143^**^	1				
4. Physical and mental health experience	0.746^**^	0.807^**^	0.152^**^	1			
5. Self-development experience	0.779^**^	0.856^**^	0.175^**^	0.921^**^	1		
6. Adaptation and satisfaction experience	0.803^**^	0.866^**^	0.163^**^	0.890^**^	0.930^**^	1	
7. Subjective well-being	0.798^**^	0.867^**^	0.168^**^	0.967^**^	0.979^**^	0.966^**^	1

^*^p < 0.05, ^**^p < 0.01, ^***^p < 0.001.

### 3.6 Hierarchical regression analysis of the impact of learning participation on subjective wellbeing among rural empty-nest and non-empty-nest elderly

#### 3.6.1 Multicollinearity test

To explore the impact of learning atmosphere, learning experience, and learning engagement on subjective wellbeing among rural empty-nest and non-empty-nest elderly groups, hierarchical regression analysis is used. Before conducting the regression analysis, it is necessary to check for multicollinearity. Multicollinearity refers to the high correlation between variables that can affect the analysis results. The condition for multicollinearity is a Variance Inflation Factor (VIF) >10.00 and an average VIF >1.00. After standardizing the hierarchical independent variables, the average VIF was 1.03, slightly above 1.00, but all variable VIFs were below 5.00, indicating no severe multicollinearity issues and allowing for hierarchical regression analysis.

#### 3.6.2 Hierarchical regression model construction

Based on the descriptive statistics and correlation analysis mentioned above, hierarchical regression is conducted separately for the entire rural elderly population, the rural empty-nest elderly population, and the rural non-empty-nest elderly population. Model 1 includes only learning variables (learning atmosphere, learning experience, learning engagement). The regression results show that both the learning atmosphere and learning experience have a significant impact on the subjective wellbeing of elderly empty nesters in rural areas. Therefore, Hypothesis 2 is validated. Model 2 adds individual demographic factors (age, gender, education level, monthly income). Model 3 further incorporates family-related demographic factors (marital status) to provide a more comprehensive reflection of the impact and variation of learning participation on subjective wellbeing among rural empty-nest and non-empty-nest elderly groups. Let (Y) be the dependent variable (subjective wellbeing), and (X1, X2…Xk) be the independent variables (elderly learning participation and its factors). The multiple linear regression model is specified as: (Yi = β0 + β1X1 + β2X2… + β*i*X*i* + ε) (see Table 6).

**Table 6:** Regression results of the impact of learning participation on the subjective wellbeing of rural elderly (*n* = 1,234).

**Variables**	**All samples**			**Empty-nest elderly sample**			**Non-empty-nest elderly sample**		
	**Model 1**	**Model 1a**	**Model 1b**	**Model 2**	**Model 2a**	**Model 2b**	**Model 3**	**Model 3a**	**Model 3b**
Learning atmosphere	0.208^***^ (7.243)	0.205^***^ (7.135)	0.205^***^ (7.147)	0.195^***^ (5.295)	0.192^***^ (5.252)	0.193^***^ (5.275)	0.208^***^ (4.501)	0.203^***^ (4.348)	0.203^***^ (4.342)
Learning experience	0.692^***^ (24.975)	0.686^***^ (24.819)	0.686^***^ (24.837)	0.723^***^ (20.024)	0.717^***^ (19.986)	0.716^***^ (19.973)	0.644^***^ (15.183)	0.644^***^ (14.872)	0.644^***^ (14.877)
Learning investment	0.023 (1.182)	0.029 (1.473)	0.029 (1.484)	0.060^*^ (2.565)	0.066^**^ (2.674)	0.065^**^ (2.615)	−0.050 (−1.570)	−0.040 (−1.214)	−0.038 (−1.124)
Age		−0.005 (−0.247)	0.002 (0.095)		0.017 (0.777)	0.023 (1.019)		−0.017 (−0.508)	−0.011 (−0.308)
Gender		0.087^**^ (2.325)	0.095^**^ (2.515)		0.080 (1.756)	0.087 (1.906)		0.074 (1.108)	0.081 (1.214)
Monthly income		0.00 (0.333)	0.004 (0.249)		−0.005 (−0.224)	−0.006 (−0.300)		0.013 (0.442)	0.012 (0.431)
Education level		−0.041^**^ (−2.865)	−0.042^**^ (−2.903)		−0.051^**^ (−2.865)	−0.051^**^ (−2.866)		−0.022 (−0.933)	−0.024 (−0.986)
Marital status			−0.065 (−1.532)			−0.063 (−1.151)			−0.933 (−0.820)
*N*	1,234	1,234	1,234	792	792	792	442	442	442
*R* ^2^	0.735	0.737	0.738	0.762	0.767	0.767	0.676	0.673	0.673
*F*	1,138.128 (sig = 0.000)	495.272 (sig = 0.000)	434.132 (sig = 0.000)	842.087 (sig = 0.000)	368.781 (sig = 0.000)	322.983 (sig = 0.000)	304.792 (sig = 0.000)	130.766 (sig = 0.000)	114.418 (sig = 0.000)

^*^p < 0.05, ^**^p < 0.01, ^***^p < 0.001.

The regression analysis of the total elderly sample reveals that the standardized multiple linear regression equations for the entire sample, the empty-nest sample, and the non-empty-nest sample are as follows:

Y (Full sample) = 0.208^*^Learning Atmosphere + 0.692^*^Learning Experience + 0.023^*^Learning Investment

Y (Empty nest group sample) = 0.195^*^Learning Atmosphere + 0.723^*^Learning Experience + 0.060^*^Learning Investment

Y (Non-empty nest group sample) = 0.208^*^Learning Atmosphere + 0.644^*^Learning Experience – 0.050^*^Learning Investment

The results of Model 1 show that both learning atmosphere and learning experience have a significant positive impact on the subjective wellbeing of the overall elderly population (*F* = 1238.128, *P* < 0.001), with an (*R*^∧^2) of 0.735, indicating that 73.5% of the variance in subjective wellbeing can be explained. According to the regression results for the rural empty-nest elderly sample and the rural non-empty-nest elderly sample, the positive impact of learning experience and learning engagement on the subjective wellbeing of the empty-nest elderly is greater than that for non-empty-nest elderly learners. Conversely, only the learning atmosphere factor has a more significant positive effect on the subjective wellbeing of the non-empty-nest elderly compared to the empty-nest elderly. In summary, learning participation has a more positive effect on the subjective wellbeing of rural empty-nest elderly individuals compared to rural non-empty-nest elderly individuals.

To further test the hypothesis, individual characteristics and family-related variables were sequentially added in Models 2 and 3. Learning engagement's impact on the overall elderly population's subjective wellbeing increased, while the effects of learning atmosphere and learning experience decreased, though they remained significantly positive. For the rural empty-nest elderly sample, the inclusion of individual factors such as age, gender, monthly income, and education level significantly enhanced the impact of learning engagement on subjective wellbeing, but reduced the impact of learning atmosphere and experience. With the addition of family factors, the effect of learning atmosphere improved, while the effects of learning experience and engagement declined. For the rural non-empty-nest elderly sample, after adding individual factors, the impact of all learning dimensions on subjective wellbeing decreased, with learning engagement having a negative effect. However, after incorporating family factors, the effects of learning atmosphere, learning experience, and learning engagement on subjective wellbeing increased. Despite the changes in the impact of learning participation on subjective wellbeing with the inclusion of individual and family factors, the positive effects of learning experience and learning atmosphere on the subjective wellbeing of rural empty-nest elderly remain higher than those for rural non-empty-nest elderly.

The results indicate that, both before and after accounting for individual and family factors, the positive effects of learning atmosphere and learning experience on the subjective wellbeing of rural empty-nest and rural non-empty-nest elderly populations are significant, with a high explanatory power for subjective wellbeing. Learning experience and learning engagement have higher coefficients for subjective wellbeing in vulnerable elderly groups compared to non-vulnerable elderly learners. Furthermore, learning engagement negatively impacts the subjective wellbeing of rural non-empty-nest elderly individuals. Overall, the analysis of Models 1, 1a, 1b; 2, 2a, 2b; and 3, 3a, 3b shows that the positive impact of learning participation on the subjective wellbeing of rural empty-nest elderly is more pronounced. Thus, Hypothesis 3 is supported.

## 4 Research conclusions and discussion

First, there is a significant difference in subjective wellbeing between rural empty-nest elderly and non-empty-nest elderly, with the former experiencing lower levels of subjective wellbeing. Empty-nest elderly often face greater psychological and social pressures, which can impact their quality of life and attitude toward learning. Firstly, empty-nest elderly lack the companionship and support of family members, leading to feelings of loneliness and helplessness (Zhang, 2023). In rural areas, family relationships typically play a crucial role in social support (Wang, 2024). However, as children migrate and work elsewhere, the support network of empty-nest elderly deteriorates, leaving them without effective support and comfort during difficulties or emotional needs. In contrast, non-empty-nest elderly usually rely on family members' companionship and care, helping them maintain a more positive emotional state and higher subjective wellbeing. Secondly, empty-nest elderly are more prone to negative emotions such as loneliness, depression, and irritability, and have weaker emotional control, adversely affecting their mental health (Fung and Chan, 2008). Research shows that 60% of empty-nest elderly in rural China have psychological issues, with 20% developing conditions needing medical attention (Liang et al., 2020). Feelings of loneliness and helplessness exacerbate negative emotions and impact their subjective wellbeing. Additionally, psychological problems lead to reduced interest, decreased activity, and weakened sense of autonomy (McCusker et al., 2016). Therefore, mental health issues affect their attitude toward and participation in learning activities, leading to a more negative mindset. Moreover, empty-nest elderly face issues of role degradation in social identity (Levy et al., 2002). The family plays a critical role in social life, and the empty-nest status causes elderly individuals to lose their central role within the family, affecting their self-identity and social role. In contrast, non-empty-nest elderly often play more active roles, such as caring for grandchildren or participating in family decisions, which helps them establish a more stable social identity and enhance their subjective wellbeing. Finally, empty-nest elderly face reduced social activities due to fewer family members or lack of a partner. Social activities are vital for maintaining social connections and receiving support; the empty-nest status limits their social range, leading to greater social isolation (Courtin and Knapp, 2017).

Second, participation in learning significantly enhances the subjective wellbeing of rural empty-nest elderly, with a notable positive correlation between various dimensions of learning participation and subjective wellbeing. The different dimensions of learning participation positively impact the subjective wellbeing of rural empty-nest elderly by offering social interaction and support, active engagement, and self-improvement opportunities, thereby improving life satisfaction and happiness. Firstly, the learning environment refers to the learning conditions and state of other members within the individual's learning context (Hou, 2021), directly affecting their learning experience. A positive learning environment includes support and encouragement, interaction, and communication with others, which boosts self-confidence and enhances happiness. Secondly, learning experience involves the individual's perception, view, and understanding of the learning environment (Entwistle and Ramsden, 1982). The positive learning experience comes from the sense of achievement and self-fulfillment gained during learning. Social support theory suggests that perceived social support can mitigate the negative impacts of stress (Cohen and Kay, 1984). Positive learning experiences enhance self-awareness and self-esteem, leading to higher life satisfaction and happiness (Diener, 2000). Positive psychology focuses on individual strengths, happiness, and wellbeing, emphasizing the cultivation of positive emotions, personal traits, and life meaning to boost happiness. Overcoming learning challenges or acquiring new skills provides a sense of accomplishment that directly enhances happiness. Lastly, learning engagement refers to the effort, time, and energy invested in learning activities (Schaufeli et al., 2002). Research indicates that high learning engagement improves learning outcomes and sense of achievement, thus enhancing subjective wellbeing (Fredricks et al., 2004). The degree of engagement in learning is crucial for boosting subjective happiness. When elderly individuals are passionate about learning and actively participate, they generally feel more satisfied and happy. This engagement provides a sense of purpose and value, increasing their expectations and hopes for the future.

Third, compared to non-empty-nest elderly in rural areas, learning participation has a more pronounced positive effect on rural empty-nest elderly. After controlling for individual (gender, age, monthly income, education) and family (household registration, marital status, living conditions) factors, hierarchical regression analysis reveals that the two dimensions of learning participation—learning experience and learning engagement—explain a significant portion of the subjective wellbeing of rural empty-nest elderly. Learning experience and investment, as key dimensions of learning engagement, play a vital role in enhancing the subjective wellbeing of rural empty-nest elderly. Firstly, compared to non-empty-nest elderly, the migration of children, the death of spouses, or weakened community relationships often lead rural empty-nest elderly to feel loneliness and social deficiency, with limited resources in rural areas exacerbating this feeling. Thus, participation in educational activities helps empty-nest elderly establish new social connections and support networks. Through learning, they can build relationships with other learners and teachers, increasing social interaction and alleviating loneliness. This social interaction helps fill the social void created by their empty-nest status. In contrast, non-empty-nest elderly typically have more family members around, and their social needs may be relatively met, making the impact of learning participation on their subjective wellbeing less pronounced. Secondly, self-determination theory posits that autonomy and intrinsic motivation in one's actions are key to achieving happiness (Li, 2023). Compared to non-empty-nest elderly, empty-nest elderly, free from caregiving burdens, can devote more energy to learning, adjusting their lifestyle and finding new goals and pleasures in late life. Their efforts and engagement in learning satisfy intrinsic motivation, enhancing learning outcomes and enriching life experiences, which increases self-fulfillment and happiness.

## 5 Limitations and future directions

This study has certain limitations. Firstly, the scope is limited as it focuses only on elderly learners aged 50 and above in specific districts within Z Province's N City, which restricts the generalizability of the results. Future research should broaden the scope to include a wider geographical area, and samples from various socio-economic backgrounds to enhance the applicability and reliability of the findings. Secondly, the use of quantitative methods may overlook individual experiences, emotions, and perspectives, potentially affecting a comprehensive understanding of subjective wellbeing. Future studies should adopt a mixed-methods approach to gain a more thorough and nuanced understanding, including in-depth exploration of individual experiences and viewpoints. Lastly, using electronic questionnaires might exclude some rural empty-nesters who are unfamiliar with or unable to access electronic devices, potentially causing sample bias. Future research should employ diverse survey methods, such as face-to-face interviews or telephone surveys, to ensure sample diversity and representativeness and better capture the subjective wellbeing of rural empty-nesters.

## 6 Innovative evaluation

The study demonstrates innovation in three ways. First, the research employed a quantitative research method, utilizing random sampling and data analysis to systematically measure the impact of learning participation on the subjective wellbeing of rural empty-nest elderly. This approach provides new insights and a foundation for quantitative research in this field. Second, the research perspective is innovative. Traditionally, researchers are more inclined to explain the influencing factors of subjective wellbeing of the elderly from the aspects of economy, health, and social support, while relatively little attention has been paid to learning participation. Therefore, by introducing learning participation as an independent variable into the research framework, it provides new perspectives and ideas for exploring the influencing factors of subjective wellbeing of rural elderly groups. Finally, this study's content innovation lies in treating learning engagement as a variable and exploring its impact on the subjective wellbeing of rural empty-nest elderly. It proposes a new approach of enhancing learning engagement to improve elderly individuals' psychological wellbeing, social integration, and self-identity, emphasizing the potential role of learning activities in enhancing the quality of life and happiness of elderly people.

## Data Availability

The raw data supporting the conclusions of this article will be made available by the authors, without undue reservation.
